# Supplementary material for: Effectiveness and Safety of Ayurvedic Medicines in Type 2 Diabetes Mellitus Management: A Systematic Review and Meta-Analysis
Source: Front Pharmacol. 2022 Jun 8;13:821810. doi: 10.3389/fphar.2022.821810 (PMC9213670; doi:10.3389/fphar.2022.821810)
Supplement: Supplementary file 1 [file DataSheet1.zip › Supplementary Material/Forest and Funnel Plots/Trigonella foenum-graecum/Fasting insulin.pdf]

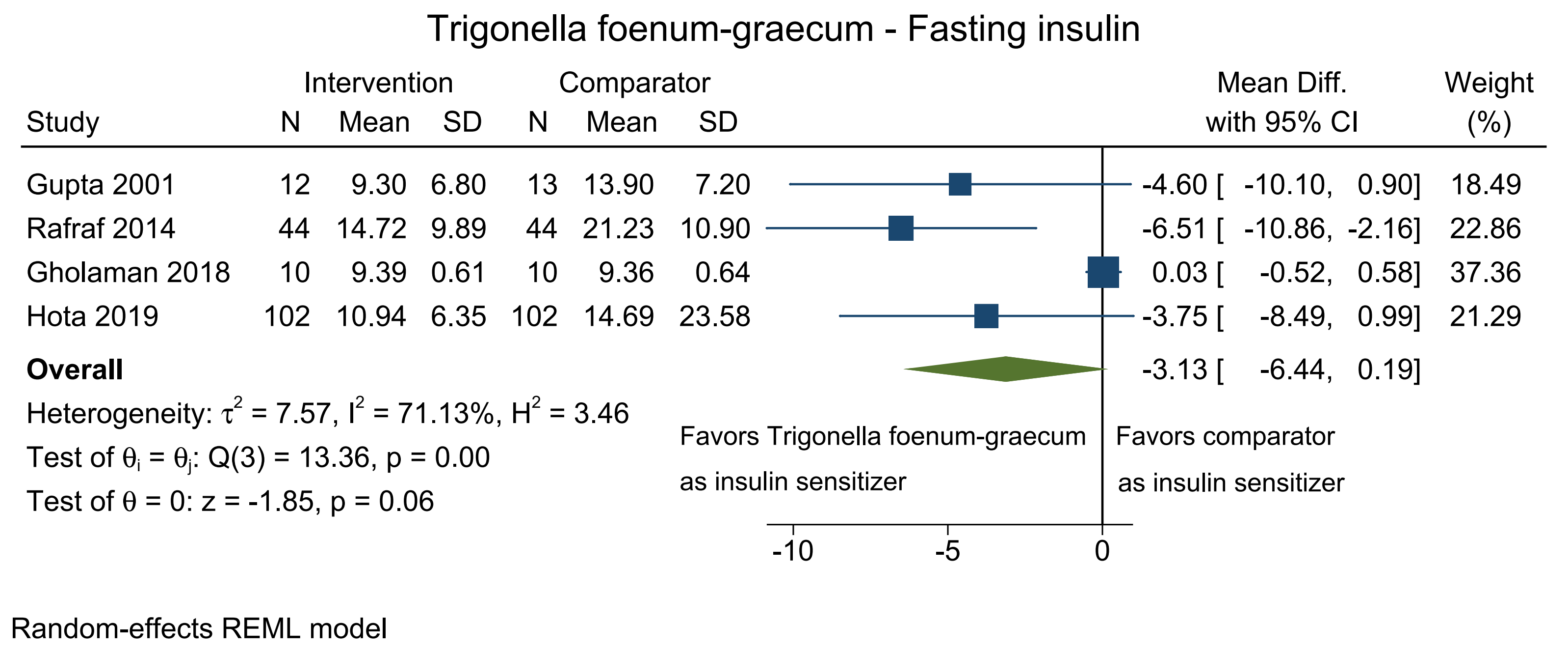
Trigonella foenum-graecum - Fasting insulin
Intervention
Comparator
Study
N
Mean
SD
N
Mean
SD
Mean Diff. with 95% CI
Weight (%)
Gupta 2001 12 9.30 6.80 13 13.90 7.20 -4.60 [-10.10, 0.90] 18.49
Rafraf 2014 44 14.72 9.89 44 21.23 10.90 -6.51 [-10.86, -2.16] 22.86
Gholaman 2018 10 9.39 0.61 10 9.36 0.64 0.03 [-0.52, 0.58] 37.36
Hota 2019 102 10.94 6.35 102 14.69 23.58 -3.75 [-8.49, 0.99] 21.29
Overall -3.13 [-6.44, 0.19]
Heterogeneity: τ² = 7.57, I² = 71.13%, H² = 3.46
Test of θi = θj: Q(3) = 13.36, p = 0.00
Test of θ = 0: z = -1.85, p = 0.06
Favors Trigonella foenum-graecum as insulin sensitizer
Favors comparator as insulin sensitizer
-10
-5
0
Random-effects REML model
